# Supplementary material for: A Benzothiadiazole-Based Self-Assembled Cage for Cadmium Detection
Source: Molecules. 2023 Feb 15;28(4):1841. doi: 10.3390/molecules28041841 (PMC9967857; doi:10.3390/molecules28041841)
Supplement: Supplementary file 1 [file molecules-28-01841-s001.zip › molecules-2217342-supplementary.pdf]

Supporting Information

Benzothiadiazole-based self assembled cage for cadmium detection

Zong-Cheng Wang[1], Ying-Zi Tan[1], Hui Yu[1], Wen-Hu Bao[1], Lin-Li Tang[1] and Fei Zeng[1,*]

[1]*Hunan University of Science and Engineering, YongZhou 425199, China*

E-mail: zengfei@iccas.ac.cn

Content

1. **Experiment section..2**
2. **^{1}H NMR and ^{13}C NMR spectra of cage 1...3**
3. **^{1}H -^{1}H COSY and ^{1}H-^{1}H NOESY spectra of cage 1 ....4**
4. **Fluorescence photographs of cage 1 after additon of Cd^{2+} 5**
5. **ESI-MS spectrum of cage 1...5**
6. **^{1}H NMR spectroscopic of cage 1 after additon of Cd^{2+} 6**
7. **Reference. 6**

1. Experiment section.

General. ^{1}H NMR, ^{13}C NMR spectra were recorded on a Bruker DMX400 NMR spectrometer. Commercial reagents were used without further purification. 4, 4'-(benzothiadiazole-4,7-diyl)dibenzaldehyde **2** was prepared according to the reported literature[S1]. Electrospray ionization mass spectra (ESI-MS) were recorded on the Thermo Fisher® Exactive LC-MS spectrometer.

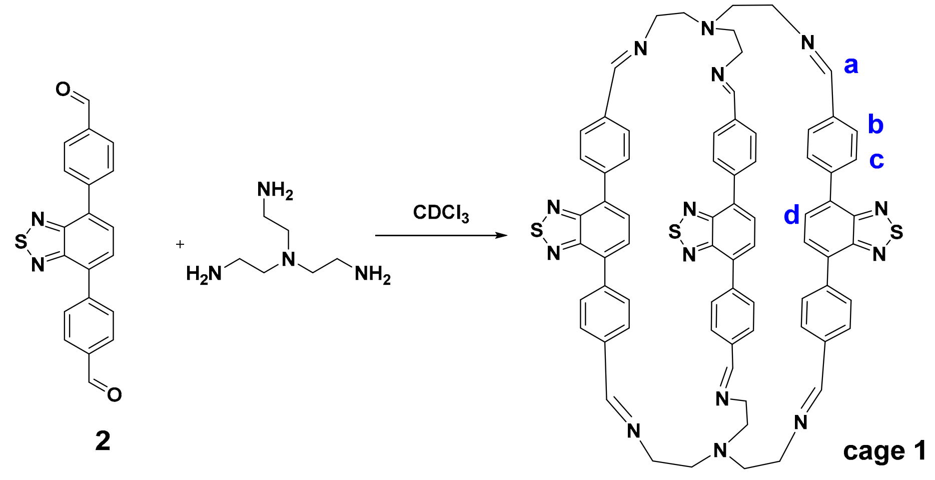

Scheme S1 Synthesis of the cage **1**

Synthesis of cage 1. The cage **1** was obtained by condensing **2** (1.24 mg, 6 mM) and tris(2-aminoethyl)amine (TREN) (0.36 mg, 4 mM) in $CDCl_3$ (0.6 mL). The solution was sealed in a NMR tube without stirring for 12h to let the system to reach the equilibrium. ^{1}H NMR (400 MHz, Chloroform-*d*) δ 8.34 (s, 6H), 7.59 (d, J = 8.0 Hz, 12H), 7.39 (d, J = 8.0 Hz, 12H), 6.71 (s, 6H), 3.91 (s, 12H), 2.90 (s, 12H). ^{13}C NMR (101 MHz, $CDCl_3$) δ 161.96, 153.58, 137.89, 136.15, 132.17, 129.05, 128.41, 127.11, 57.90, 52.22. ESI-MS cald. for $[M+Na]^+$: 1239.42. Found: 1239.15.

2. ^{1}H NMR and ^{13}C NMR spectra of cage 1

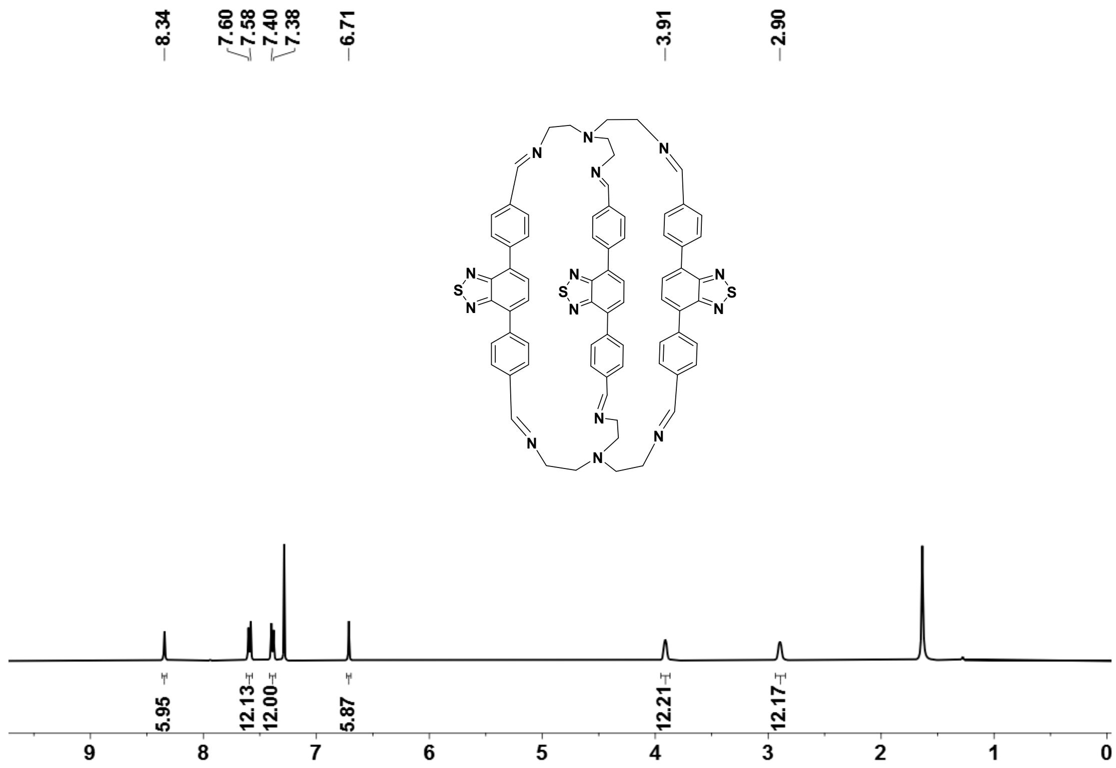

Figure S1. ^{1}H NMR spectrum (400 MHz, $CDCl_3$, 298K) of cage **1**.

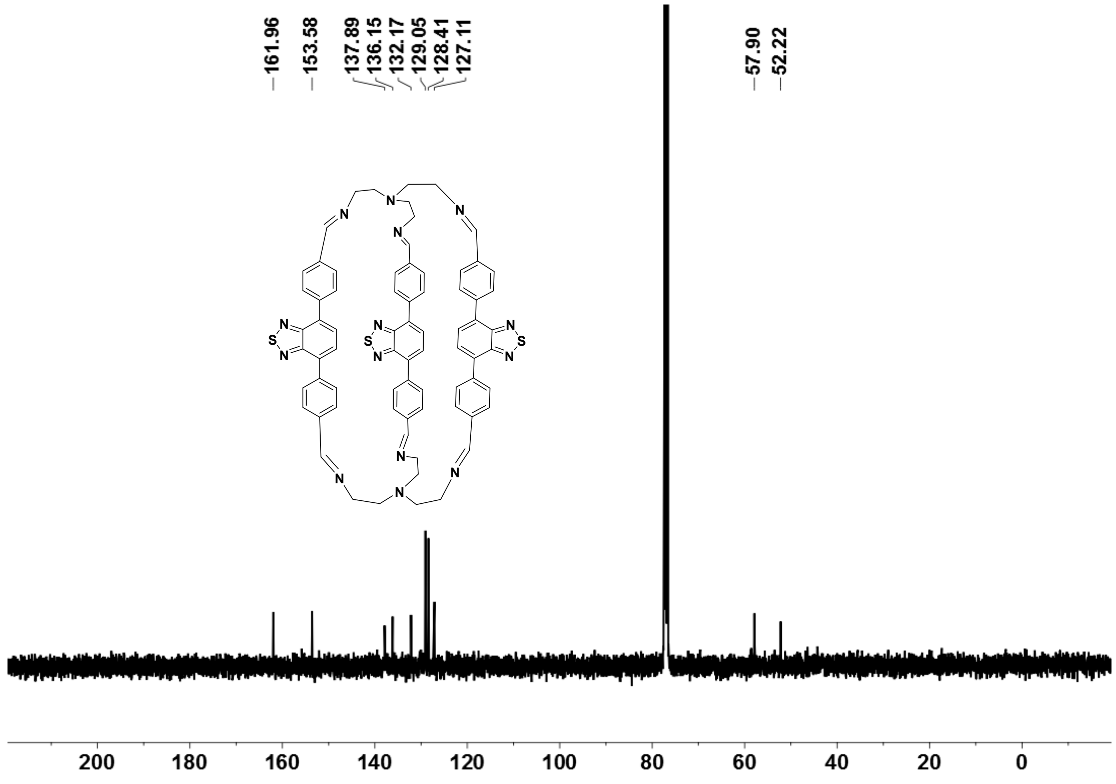

Figure S2. ^{13}C NMR spectrum (101 MHz, $CDCl_3$, 298K) of cage **1**.

3. ^{1}H -^{1}H COSY and ^{1}H-^{1}H NOESY spectra of cage 1

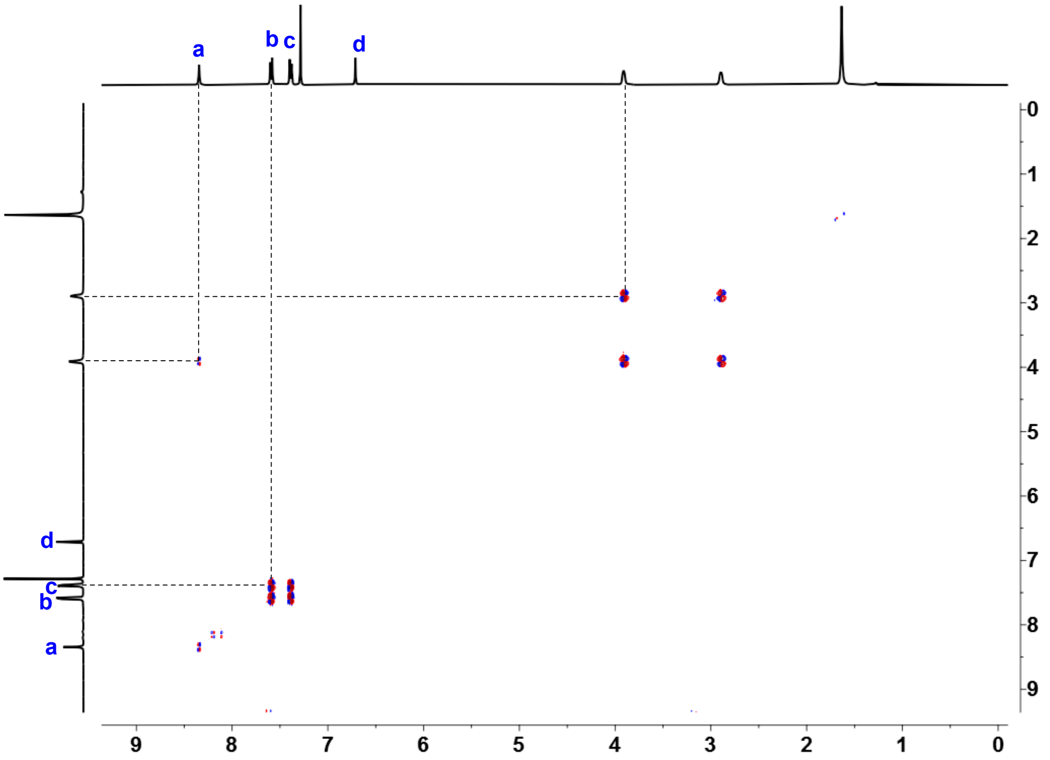

Figure S3. Partial ^{1}H-^{1}H COSY spectrum (400 MHz, $CDCl_3$, 298 K) of cage **1**.

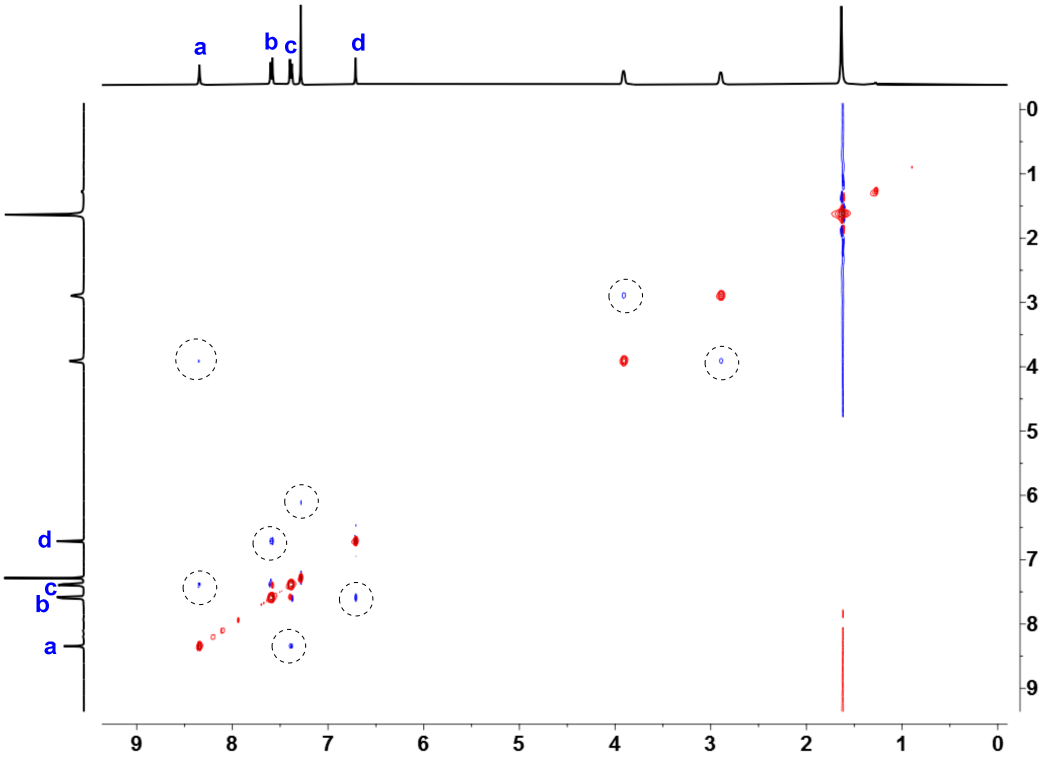

Figure S4. Partial ^{1}H-^{1}H NOESY spectrum (400 MHz, $CDCl_3$, 298 K) of cage **1**.

4. Fluorescence photographs of cage 1 after additon of Cd^{2+}

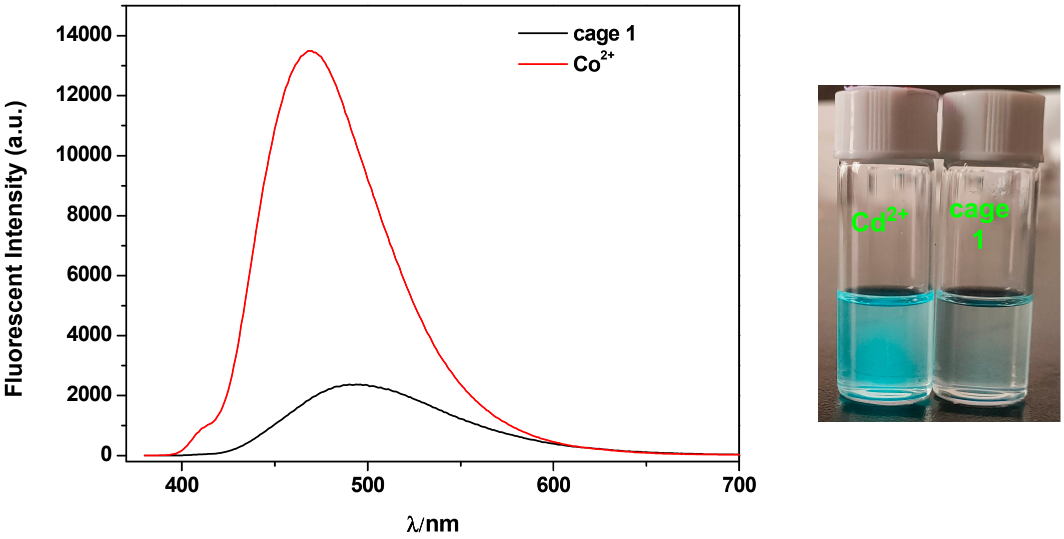

Figure S5. Fluorescence photographs of cage **1** after addition of 10 equiv of Cd^{2+}, $[1]_0 = 1.0 \times 10^{-5}$ M.

5. ESI-MS spectrum of cage 1

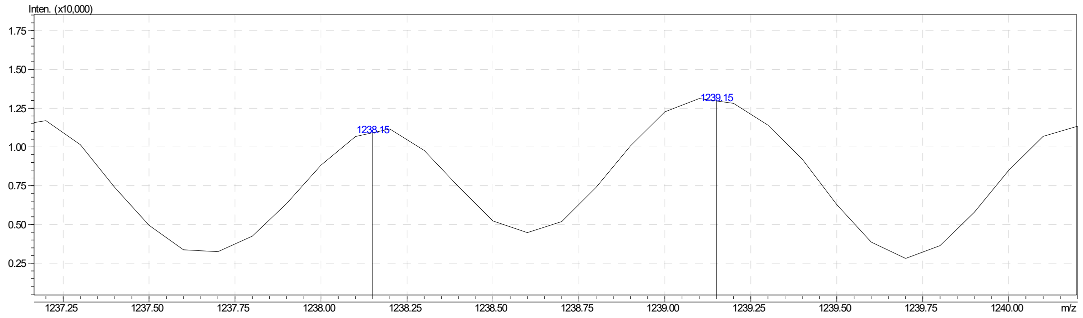

Figure S6. ESI-MS spectrum of cage **1**.

6. ^{1}H NMR spectroscopic of cage 1 after additon of Cd^{2+}

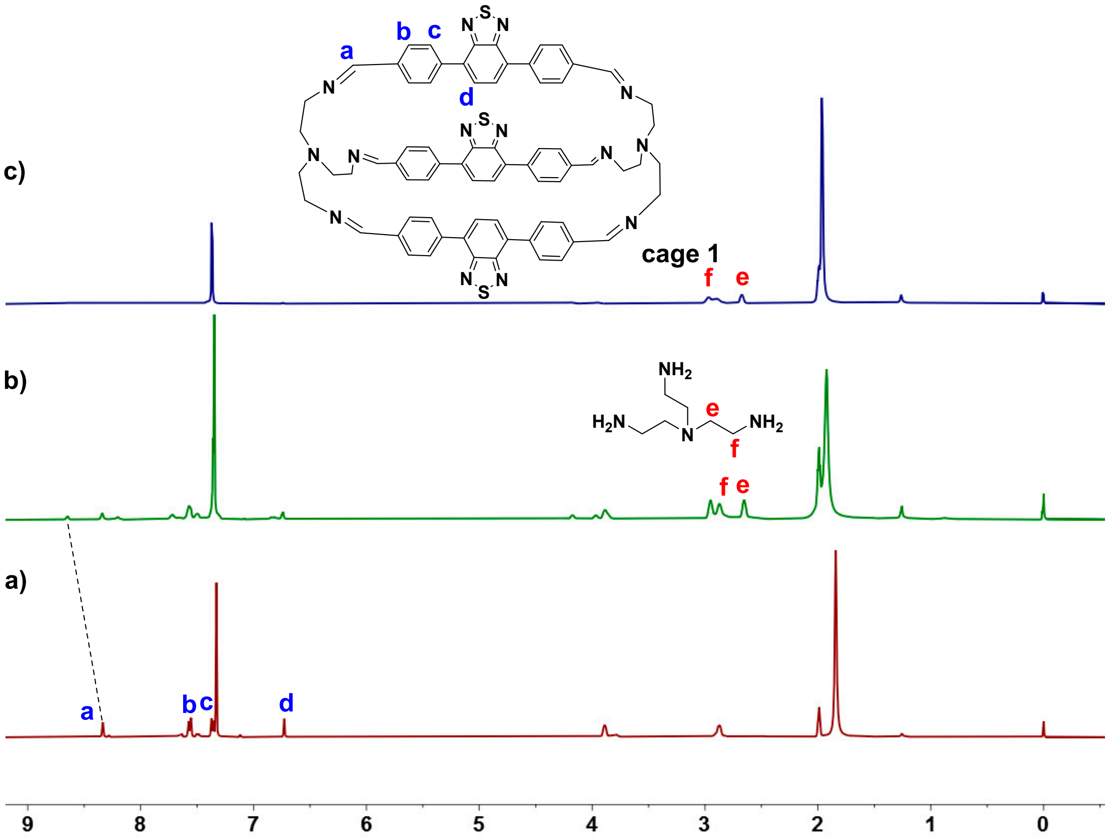

Figure S7. ^{1}H NMR spectrum (400 MHz, $CDCl_3/CD_3CN$ =10:1 , 298K) of a) cage **1**, b) **1** and 0.5 equiv of Cd^{2+}, c) **1** and 1.0 equiv of Cd^{2+}. $[\mathbf{1}]_0$ = 2.0 mM. After addition of 0.5 equiv of Cd^{2+} cation, a small amount of yellow precipitation was formed. Further increase the amount of Cd^{2+} cation can produce more precipitation.

7. Reference.

1. S1 Tang Y, Huang H, Peng B, Chang Y, Li Y, Zhong C. A thiadiazole-based covalent triazine framework nanosheet for highly selective and sensitive primary aromatic amine detection among various amines. J. Mater. Chem. A 2020; 8: 16542-16550.
